# Undetected post-traumatic stress disorder in secondary-care mental health services: systematic review

**DOI:** 10.1192/bjp.2017.8

**Published:** 2018-01

**Authors:** Stan Zammit, Catrin Lewis, Sarah Dawson, Hannah Colley, Hannah McCann, Alice Piekarski, Helen Rockliff, Jonathan Bisson

**Affiliations:** 1 MRC Centre for Neuropsychiatric Genetics and Genomics, Division of Psychological Medicine and Clinical Neuroscience, Cardiff University, Cardiff and Centre for Academic Mental Health, University of Bristol, Bristol; 2 MRC Centre for Neuropsychiatric Genetics and Genomics, Division of Psychological Medicine and Clinical Neuroscience, Cardiff University, Cardiff; 3 Centre for Academic Mental Health, University of Bristol, Bristol; 4 MRC Centre for Neuropsychiatric Genetics and Genomics, Division of Psychological Medicine and Clinical Neuroscience, Cardiff University, Cardiff; 5 Centre for Academic Mental Health, University of Bristol, Bristol; 6 MRC Centre for Neuropsychiatric Genetics and Genomics, Division of Psychological Medicine and Clinical Neuroscience, Cardiff University, Cardiff, UK

## Abstract

**Background:**

Comorbid post-traumatic stress disorder (PTSD) is associated with poorer outcomes of other disorders, but is treatable.

**Aims:**

To estimate the frequency of clinically undetected PTSD in secondary care.

**Method:**

A systematic review of studies that screened for PTSD and reported on PTSD documentation in clinical records. Frequency of undetected PTSD was estimated, and reasons for heterogeneity explored.

**Results:**

The median proportion of participants with undetected PTSD (29 studies) was 28.6% (interquartile range 18.2–38.6%). There was substantial heterogeneity, with studies conducted in the USA and those with the highest proportions of in-patients and patients with psychotic disorder reporting higher frequencies of undetected PTSD.

**Conclusions:**

Undetected PTSD is common in secondary care, even if the true value is at the lower limit of the estimates reported here. Trials examining the impact of routine screening for PTSD are required to determine whether such programmes should be standard procedure for all mental health services.

**Declaration of interest:**

None.

Identification of comorbid post-traumatic stress disorder (PTSD) in patients with other serious mental illness is of substantial importance given that this is associated with poorer clinical outcomes of these disorders,^1^^–^^4^ and that PTSD is a treatable disorder.^5^ Some studies suggest that a large proportion of patients in secondary-care mental health services with other (non-PTSD) primary diagnoses meet criteria for PTSD on screening, and that there is usually no record of PTSD in the patient's clinical records.^6^^–^^9^ If these estimates of undetected PTSD are accurate this raises a serious concern that PTSD is not adequately identified through routine clinical care pathways. Other studies, however, report much lower frequencies of undetected PTSD.^10^^–^^12^ Reasons for variation in estimates of PTSD across studies could include differences in the characteristics of the people in the study (such as gender, primary diagnosis) or of the study methodology (such as measurement or selection bias). An accurate estimate of the frequency of undetected PTSD in secondary care, and an understanding of factors associated with variation in frequency is required to determine if, and how, services should respond. In this study, we systematically review the literature to determine whether undetected PTSD is present at a prevalence that would reflect substantial clinical importance within secondary-care mental health services. For the purpose of this study we define ‘substantial clinical importance’ as presence of undetected PTSD in 10% or more of patients in secondary care, an arbitrary value but one that we believe most service providers would agree merits clinical concern. Furthermore, we also aim to determine the extent to which variation in reported estimates might be because of sample characteristics or methodological biases. To our knowledge there have not been any previous systematic reviews addressing these aims.

## Method

Our protocol (online supplement DS1 available at https://doi.org/10.1192/bjp.2017.8; not pre-registered) followed Meta-analysis Of Observational Studies in Epidemiology (MOOSE) guidelines^13^ and a PRISMA checklist was completed (online supplement DS2).

### Literature search

We (S.D.) searched the following databases from 1980 to 22 August 2016: Embase, Medline, PILOTS and PsycINFO using relevant keywords and subject headings (online supplement DS3). We (H.C., H.M., A.P. and S.Z.) hand searched reference lists of included studies to identify further relevant papers. We restricted the search to published, peer-reviewed studies, but not by study design. Only English-language studies were included.

### Selection criteria

Our inclusion criteria were: (a) participants in secondary care (i.e. specialist mental health services) with a mental illness diagnosis according to DSM or ICD criteria; (b) screened for PTSD using a measure based on DSM or ICD criteria; (c) used medical records to obtain participants’ clinical diagnoses of PTSD; (d) reported proportion of sample with PTSD on screening, and with PTSD in medical records; and (e) reported data for individuals aged 16 and over. Studies where samples were selected on the basis of (a) having a diagnosis of PTSD, (b) having a trauma history, or (c) being referred to a trauma service were excluded.

### Study selection and data extraction

We examined all titles and abstracts (S.Z., H.C. and A.P.) and obtained full texts of potentially relevant papers. Working independently and in duplicate, we (S.Z., C.L., H.R., H.C., A.P. and H.M.) read the papers to determine if they met inclusion criteria using eligibility record forms (online supplement DS4). We resolved disagreements by consensus, and extracted data independently and in duplicate.

### Quality assessment

The likely internal validity for each study was assessed (S.Z., C.L. and H.R.) based on key selected components of a risk of bias tool for prevalence studies,^14^ adapted for this review. We focused particularly on sampling strategy, response rates, and masking of screening assessment and clinical records review as the likeliest sources of bias in estimating frequency of undetected PTSD. For examination of heterogeneity, studies were also categorised into low, medium, and high risk of selection bias (online Table DS1).

### Data synthesis

The proportion of PTSD that was detected (recorded) in clinical notes was estimated as: *n* with PTSD on record/*n* with PTSD on screening. The proportion with undetected PTSD was estimated as: (*n* with PTSD on screening – *n* with PTSD on record)/sample *n*. We grouped studies together and pooled data in a meta-analysis, although such summary estimates are only useful where studies are adequately homogenous. Studies were pooled using a random-effects model (using *metaprop* in Stata version 14). Presence of publication bias was investigated by viewing funnel plots and using Egger's test.^15^ We assessed heterogeneity using the *I*^2^ statistic.^16^

We investigated sources of heterogeneity by meta-regression using the *metareg* command. We hypothesised that frequency of PTSD on screening might be higher for veterans (because of potentially greater exposure to traumas, and increased recognition by clinicians), patients diagnosed with a psychotic disorder (as the hierarchical approach to diagnosis and the phenotypic overlap between re-living experiences and hallucinations could lead to reduced identification of PTSD compared with other diagnoses), in-patients (as potentially indexing more severe illness), studies using self-report PTSD questionnaires (as lower specificity than interviews) and in studies with a greater likelihood of selection bias, and that frequency might vary by country of study (because of different rates of PTSD in different populations). We also examined, as secondary hypotheses, variation in relation to age (as greater cumulative exposure to trauma with age), gender (as PTSD is more common in women) and year of publication (as services studied in recent publications might be more aware of comorbid PTSD).

We also examined pooled estimates for specific diagnostic categories: (a) psychotic disorders, (b) affective disorders (depressive disorders and bipolar disorder), (c) substance use disorders, (d) anxiety and adjustment disorders, (e) eating disorders, and (f) personality disorders. As relatively few studies included only participants with one primary diagnosis or presented results separately for diagnostic groups, we also repeated these analyses including studies where >50% of the sample had the same diagnosis.

## Results

### Search results

**Fig. 1.**
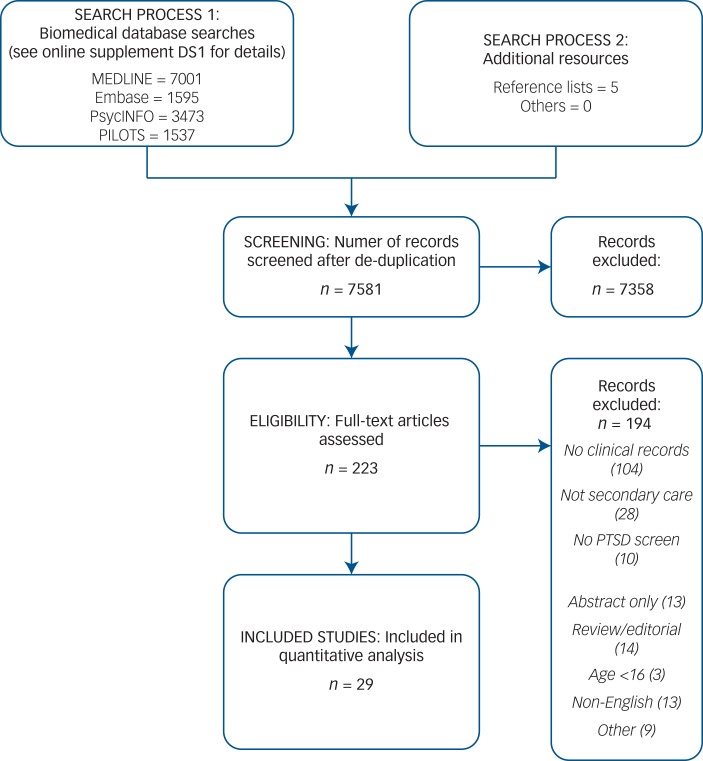
PRISMA flow diagram.

The literature search yielded 7581 references. After reading titles and abstracts, the full articles of 223 papers were assessed for eligibility, and 194 of these were excluded (PRISMA flow diagram, Fig. 1).

PTSD, post-traumatic stress disorder.

### Included studies

We included 29 studies (6412 individuals) that had data to allow us to estimate the extent of undetected PTSD in a secondary-care mental health setting (online Table DS2). Of these, 15 were based in the USA,^7^^–^^11^^,^^17^^–^^26^ 3 in each of Australia,^27^^–^^29^ the UK^6^^,^^30^^,^^31^ and the Netherlands,^32^^–^^34^ 2 in Germany,^12^^,^^35^ and 1 each in South Africa,^36^ Spain^37^ and Turkey.^38^ Three studies were in military veteran populations^7^^,^^17^^,^^18^ and 26 were in non-veterans, 1 within an incarcerated population.^23^ Most studies (*n* = 16) included individuals with a variety of mental health disorders, whereas the rest ascertained individuals with specific disorders (six with substance use disorders,^7^^,^^17^^,^^22^^,^^30^^,^^31^^,^^33^ four with psychotic disorders,^6^^,^^18^^,^^32^^,^^34^ two with mood or anxiety disorders^28^^,^^36^ and one with eating disorders^35^). The mean age of participants (28 studies) was 38.8 years (range 26.5 to 51.9 years).

### Prevalence of PTSD

The median prevalence of PTSD on screening was 33.3% (interquartile range (IQR) 23.4–40.0%), with evidence of substantial heterogeneity across studies (pooled mean 31%, 95% CI 26–36%, *I*^2^ = 93.1%) (online Fig. DS1). When we examined the prevalence of PTSD on screening within specific disorder categories the heterogeneity was substantially lower for studies with a primary diagnosis of a substance use disorder, but not for other diagnostic groups (online Fig. DS2). The mean prevalence of PTSD on screening in samples of participants with a substance use disorder was 36% (95% CI 33–40%, study *n* = 6, 793 individuals, *I*^2^ = 0%), with a psychotic disorder was 31% (95% CI 21–41%, study *n* = 6, 2994 individuals, *I*^2^ = 89.1%), with an affective disorder was 39% (95% CI 19–62%, study *n* = 3, 155 individuals, *I*^2^ = 87.4%) and with a mixture of disorders was 30% (95% CI 22–39%, study *n* = 16, 2303 individuals, *I*^2^  =  94.5%).

The median prevalence of PTSD diagnoses in clinical records was 2.3% (IQR 1.1–4.5%), with evidence of substantial heterogeneity (mean 3%, 95% CI 2–4%, *I*^2^  =  85.3%). Of those with PTSD on screening, median level of detection (i.e. recorded in notes) was 11.5% (IQR 2.8–19.4%), with substantial heterogeneity across studies (mean 10%, 95% CI 6–16%, *I*^2^  =  86.6%).

### Undetected PTSD

**Fig. 2:**
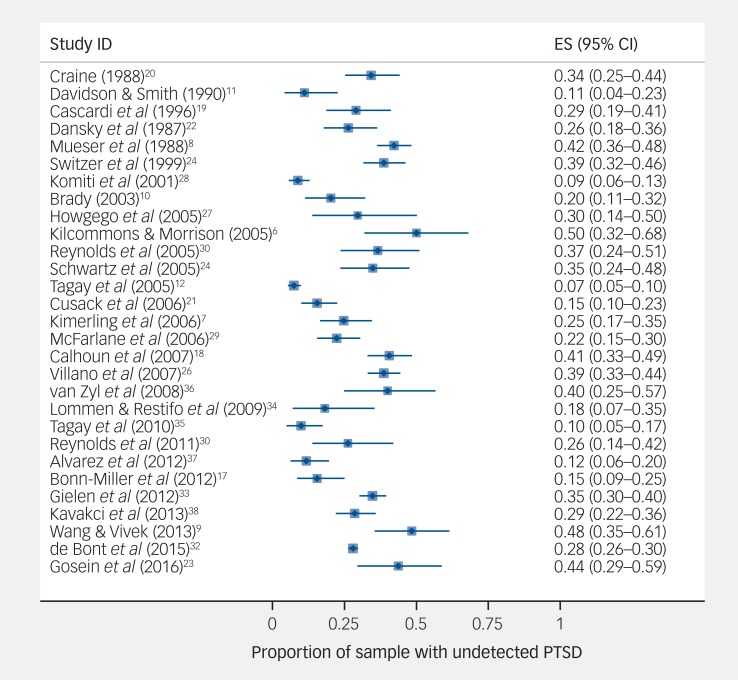
Prevalence of undetected post-traumatic stress disorder (PTSD).

The median proportion of participants within each study that had undetected PTSD was 28.6% (IQR 18.2–38.6%), with evidence of substantial heterogeneity across studies (mean 27%, 95% CI 22–32%, *I*^2^ = 93.3%) (Fig. 2). There was no evidence of publication bias arising from an absence of small studies with lower proportions of undetected PTSD (Egger test (29 studies), *P* = 0.743).

ES, effect size.

**Fig. 3:**
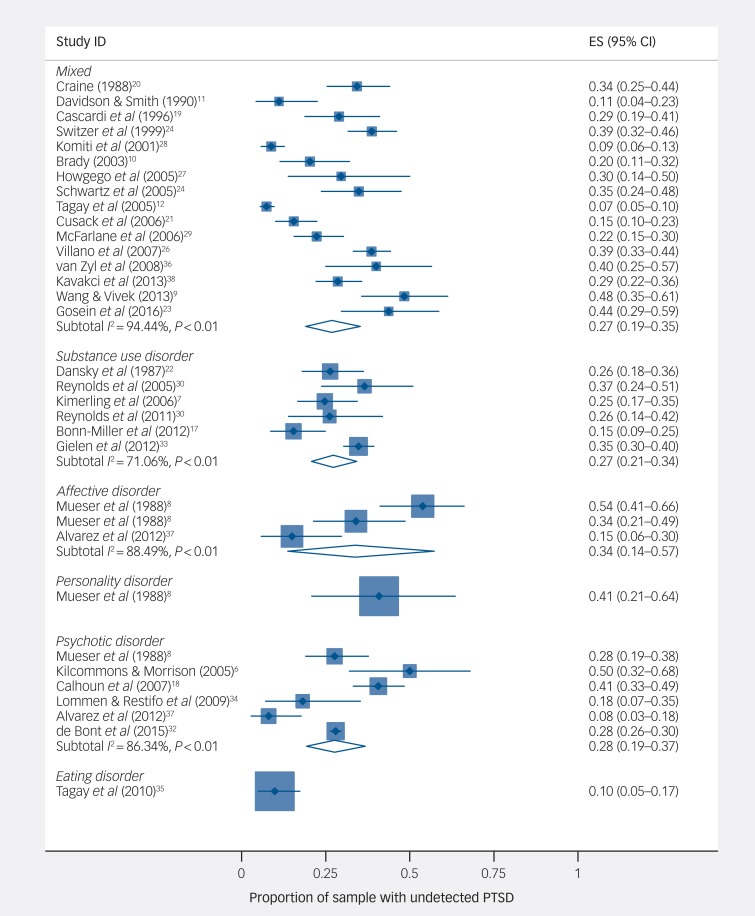
Proportion of sample with undetected post-traumatic stress disorder (PTSD), by diagnostic subgroup.

The mean prevalence of undetected PTSD in participants with psychotic disorders was 28% (95% CI 19–37%, study *n* = 6, 2994 individuals, *I*^2^ = 86.3%), with substance use disorders was 27% (95% CI 21–34%, study *n* = 6, 793 individuals, *I*^2^ = 71.1%), with affective disorders was 34% (95% CI 14–57%, study *n* = 3, 155 individuals, *I*^2^ = 88.5%), and for samples with a mixture of disorders was 27% (95% CI 19–35%, study *n* = 16, 2303 individuals, *I*^2^ = 94.4%) (Fig. 3).

ES, effect size.

Results were similar when we included studies where more than half of participants had the same diagnosis (online Fig. DS3). In a stratified analysis there was no evidence that undetected PTSD was less common when restricting studies to those using a structured interview for screening for PTSD (median proportion 26.3%, IQR = 20.3–38.6%) compared with those using a self-report questionnaire (median proportion 28.8%, IQR = 15.5–40.6%).

### Exploring heterogeneity

Given the strong evidence of heterogeneity, the overall estimates of undetected PTSD are not particularly informative, and we therefore explored potential sources of heterogeneity (Table 1 and online Table DS3).

**Table 1:** Meta-regression of primary variables explaining variation in frequency of undetected post-traumatic stress disorder (PTSD)

	Unadjusted		Adjusted^b^	
Variable^a^	β (95% CI)	*P*	β (95% CI)	*P*
Bias risk (low)	−0.02 (–0.09 to 0.05)	0.545	−0.02 (–0.08 to 0.04)	0.473
Country	0.09 (–0.01 to 0.19)	0.050	0.16 (0.07 to 0.25)	0.001
In-patients	0.05 (–0.01 to 0.11)	0.072	0.04 (–0.00 to 0.09)	0.063
Psychosis	1.05 (–0.43 to 2.53)	0.156	1.44 (0.45 to 2.42)	0.006
PTSD tool	−0.01 (–0.11 to 0.10)	0.917	0.00 (–0.09 to 0.08)	0.979
Veterans	0.01 (–0.16 to 0.18)	0.886	−0.09 (–0.22 to 0.05)	0.202

a. Bias risk (low) – coded as non-low (0), medium (1), low (2); country – coded as USA (1) *v.* rest (0); in-patients – coded as out-patients only (0), mixed (1), in-patients only (2); psychosis – per 10% increase in sample with a psychotic disorder; PTSD tool – coded as interview (0) *v.* questionnaire (1); veterans – coded as veterans (1) *v.* non-veterans (0).

b. Adjusted for other variables in the table (residual *I*^2^ = 5.6%)

#### PTSD on screening

Meta-regression showed strong evidence that levels of PTSD on screening varied across country of origin (*P* = 0.005, residual *I*^2^ = 19.6%), with estimates being higher in studies from the USA (USA *v.* rest: difference 13%, 95% CI 4–22%, *P* = 0.007). There was very weak evidence that PTSD on screening was higher in studies with higher proportions of older participants (*P* = 0.078) and men (*P* = 0.071), but little evidence that other variables explained heterogeneity.

#### Clinical detection of PTSD

There was some evidence that PTSD, if present on screening, was less likely to have been detected in samples with a higher proportion of patients with a psychotic disorder (*P* = 0.024), and more likely to have been detected in samples of veterans (*P* = 0.053) and studies that used interviews to screen for PTSD (*P* = 0.068). There was little evidence that variation in detection of PTSD was associated with the other variables examined.

#### Undetected PTSD

There was some evidence from meta-regression that levels of undetected PTSD were higher in studies from the USA (*P* = 0.050) and in studies on in-patients (*P* = 0.072), but little evidence that levels were higher in studies with proportions of patients with a psychotic disorder (*P* = 0.156). However, in a multivariable model including the primary explanatory variables, there was strong evidence that undetected PTSD was higher in studies from the USA (*P* = 0.001) and in samples with greater proportions of patients with a psychotic disorder (*P* = 0.006).

### Quality assessment

A total of 14 of the 29 studies described either random sampling or sampling of consecutive admissions or patient contacts (online Table DS3). Of these 14 studies, 9 reported response rates, 5 of which included data from 75% or more of eligible patients.

Masking of researchers to PTSD histories in clinical records was reported for 3 of the 16 studies^25^ that relied on research interviewers to determine the presence of PTSD on screening, whereas 13 studies used self-report screening measures. Masking of researchers to PTSD screening results when searching the clinical records for PTSD diagnoses was reported for 2 of the 29 studies,^10^^,^^25^ and 1 study excluded participants with PTSD in their clinical records at study entry.^36^

Estimates of PTSD on screening were lower for studies with the lowest risk of bias in a stratified analysis, although confidence intervals overlapped substantially (lowest risk 24%, 95% CI 12–40%; intermediate risk 31%, 95% CI 22–41%; highest risk 35%, 95% CI 28–42%). A similar pattern was observed for estimates of undetected PTSD (lowest risk 21%, 95% CI 9–36%; intermediate risk 29%, 95% CI 19–39%; highest risk 28%, 95% CI 22–33%).

## Discussion

In this systematic review we found the frequency of PTSD on screening was very high in samples of patients ascertained through secondary-care services. Although there was substantial heterogeneity in estimates, precluding any useful interpretation of a summary meta-analytic estimate, the IQR across studies ranged from 23 to 40%, indicating that a substantial proportion of patients in secondary-care mental health services met criteria for PTSD in most studies to date.

The frequency of PTSD on screening was high, whereas the frequency with which PTSD was documented in the clinical records was low. Our results indicate that in the majority of cases, mental health clinicians fail to recognise PTSD in their patients. Furthermore, given how common PTSD was on screening, the failure of clinical recognition of this disorder was one that potentially affected a substantial proportion of all patients under the care of specialist mental health services.

### Possible explanations

This interpretation of our results depends on a number of key assumptions. First, we are assuming that a diagnosis of PTSD would have been noted in the clinical records had mental health staff identified this as being present. Although it is possible clinicians may have been aware of a PTSD diagnosis but deemed it as irrelevant to the patient's current presentation and not worthy of recording, this seems unlikely. A second, and perhaps more critical assumption, is that the screening tools used to identify PTSD in these studies were valid and did not substantially overestimate the presence of PTSD. A total of 13 studies used self-report questionnaire measures, and all measures used have been validated, with good specificity against the Clinician-Administered PTSD Scale (CAPS) that is considered to be the gold-standard for assessment of PTSD (0.86 for the PTSD checklist (PCL);^39^ 0.93 for the Trauma Screening Questionnaire (TSQ);^40^ 0.71 for the PTSD symptom scale – self report version (PSS-SR);^41^ 0.71 for Self-Report Inventory for PTSD (SRIP)^42^). More relevant, however, is the positive predictive values (PPVs) of these instruments. The PCL, as used by some studies in this review, had a PPV of 0.8 (cut-off score 50) in a sample where 45% of participants had PTSD^39^ and a PPV of 0.7 in another study with a PTSD prevalence of 39%.^43^ Similarly the PPV for the TSQ (6+ items cut-off) was 0.86 in a study with a PTSD prevalence of 34%,^40^ for the SRIP (cut-off 52) was 0.71 with a PTSD prevalence of 47%,^44^ and for the PSS-SR was 0.64 with a PTSD prevalence of 43%.^45^

The true proportion of PTSD in these samples if screened with a gold-standard assessment might therefore be slightly lower than that estimated in these studies, although it is unlikely to be substantially lower. Consistent with this, although only 2.1% of the sample in one study had a diagnosis of PTSD in their clinical records,^33^ only a further 2.7% had a record of ‘possible PTSD’, making minimal difference to the estimates for undetected PTSD (34.8% compared with 32.2% respectively). Only three studies in our review used the CAPS, however, many studies used other diagnostic interviews that are also likely to be robust measures of PTSD. Furthermore, there was little evidence from the meta-regression that the type of PTSD assessment tool was associated with explaining heterogeneity in the frequency of PTSD on screening.

The third, and final, assumption is that the studies are not biased in a way that leads to substantial overestimates of undetected PTSD. We assessed the quality of these studies, focusing particularly on the strategy for sampling and response rates as being the most likely sources of selection bias. Studies that did not sample all consecutive admissions, or a random sample of patients, may have recruited individuals with an increased likelihood of having experienced a traumatic event, and consequently of having PTSD; for example, patients with a known trauma history may have been selected for recruitment by researchers/clinicians, or may have been more likely to participate. Such scenarios could lead to overestimates in the prevalence of PTSD on screening, and hence undetected PTSD in our review. Most studies were susceptible to such selection bias, and PTSD on screening and undetected PTSD were lower in studies rated with lowest risk of bias. However, there was little evidence from the meta-regression that selection bias risk was associated with heterogeneity in prevalence of undetected PTSD.

Although selection bias might have led to overestimates of PTSD on screening, there are also a number of reasons why prevalence of undetected PTSD may have been underestimated. Absence of masking of researchers to clinical diagnosis when conducting screening interviews, and to screening outcome when retrieving clinical records, introduces the possibility of information bias that would most likely lead to underestimates of undetected PTSD. Furthermore, in two studies underestimates of undetected PTSD may have been particularly likely. One study excluded patients with high scores on the Brief Psychiatric Rating Scale,^37^ thus potentially excluding individuals with greater likelihood of PTSD given the association between comorbid PTSD and illness severity. In the other, screening results were entered into the clinical notes,^22^ potentially influencing discharge diagnoses.

### Heterogeneity

The strongest characteristic associated with prevalence of PTSD on screening and of undetected PTSD was country, with studies from the USA having substantially higher levels of PTSD than other countries. This is consistent with population-based studies of PTSD that report lifetime prevalence of 8% in the USA^46^ compared with 1.9% in Europe.^47^ Explanations for differences across countries are almost certainly complex and likely to involve cultural dynamics and historical context, as well as variation in exposure to interpersonal violence and other sources of trauma.^48^^,^^49^ PTSD was also less likely to be detected in patients with psychotic disorders. One explanation for this is that symptoms of PTSD can be interpreted as psychotic, particularly if severe; for example, re-living experiences, especially during dissociative states, can be classed as hallucinations, and altered beliefs about safety and trust characteristic of severe PTSD can be experienced with delusional intensity.

### Strengths and limitations

We had a comprehensive search strategy in terms of the search terms and databases searched to ensure we identified relevant studies, however, we excluded studies that were not published in the English language, and this may have led to some studies being missed. Furthermore, although we took a rigorous approach to address heterogeneity in our results the number of studies in these analyses meant that power may have been limited to identify factors that had smaller effects on frequency of undetected PTSD. Although the degree of heterogeneity precluded us from deriving a meta-analytical estimate for undetected PTSD, nevertheless estimates from 22 of the 29 studies were consistent with at least 10% of patients having undetected PTSD (our *a priori* definition of clinical importance) as judged by the bounds of the confidence intervals.

Finally, the ability of a systematic review to inform evidence depends upon the quality of the included studies. A number of studies had potential for selection bias, although limiting studies to those with low risk of bias did not alter the conclusions of our findings. Furthermore, although the use of self-report questionnaires as screening instruments may be viewed as an important limitation of many of the included studies in that they might overestimate the frequency of undetected PTSD, there was no evidence that the frequency of undetected PTSD was any lower when restricting our analyses to studies that used only structured interviews for screening.

### Implications

If, as suggested by our IQR, 18 to 39% of patients in secondary-care mental health services have undetected PTSD, this has important implications for clinical services. Increased training of mental health staff in identifying symptoms of PTSD, and perhaps particularly in distinguishing re-living experiences from psychotic symptoms, seems warranted.

Presence of comorbid PTSD is associated with poorer clinical outcomes for a number of disorders. More importantly, it is possible that for some patients, the disorder being treated by clinicians (for example depression, agoraphobia, obsessional–compulsive disorder, addictions, psychosis) is secondary to PTSD; failure to recognise and treat the underlying PTSD can lead to failure in recovery from these secondary disorders. This is avoidable, as PTSD is a treatable disorder.^5^

Concerns about high levels of undetected PTSD have been voiced for approximately two decades,^8^^,^^22^^,^^25^ yet many healthcare providers do not appear to have taken adequate steps to address this problem, as evidenced by the more recent publications in our review. There is clearly a need for more robust methodology to more accurately determine the extent to which PTSD goes undetected within clinical services, and to determine the likely cost to individuals, service providers and society that might serve as drivers to motivate change. However, even if the true value of undetected PTSD is at the lower limit of the IQR reported here, there is a clear need to trial a PTSD screening programme to examine its feasibility and impact on clinical outcomes. If shown to improve patient outcomes and be cost-effective, such screening programmes should become a standard part of secondary-care mental health services in the future.

## Supplementary material

For supplementary material accompanying this paper visit https://doi.org/10.1192/bjp.2017.8. click here to view supplementary material
