# Supplementary material for: Undetected post-traumatic stress disorder in secondary-care mental health services: systematic review
Source: Br J Psychiatry. 2018 Jan;212(1):11–8. doi: 10.1192/bjp.2017.8 (PMC6457163; doi:10.1192/bjp.2017.8)
Supplement: Supplementary file 1 [file S0007125017000083sup001.zip › editedZammit et al online supplement DS1.docx]

**Online Supplement DS1**

**Systematic Review Protocol April 2015**

**Title**

What is the prevalence of undetected PTSD in secondary care mental health services?

**Objectives**

To discover the rate of undetected PTSD in adults with a diagnosis of another mental illness.

**Methods**

**Inclusion/exclusion criteria**

***Types of Studies***

We will use studies which have screened for PTSD and report prevalence of PTSD independent of screening, in order to estimate the prevalence of a given sample. Only peer reviewed studies, to ensure a high standard of methodology, and published articles will be considered. Also, only studies published from 1980 to present will be included, as PTSD was not defined as a mental disorder in DSM or ICD before this point. Studies not written in English will not be included.

[*Amendment to protocol August 2015:* Include all languages in search (as long as abstract is in English) and then see whether possible to get people to extract relevant data if relatively few in non-English language identified]

***Types of Participants***

*Age*

This review will only consider adults aged 16 and over.

*Diagnosis*

All participants will be currently suffering from a mental illness as diagnosed according to DSM-III (APA 1980), DSM-IIIR (APA 1987), DSM-IV (APA 2000), DSM-V (APA 2013), ICD-9 (WHO 1979), or ICD-10 (WHO 1992) criteria, via a DSM or ICD derived clinical diagnosis. PTSD will be diagnosed either by a clinician administering a structured interview or by using a DSM or ICD tool, such as Impact of Event Scale, Trauma Screening Questionnaire or PTSD Checklist questionnaires. No limitations will be put on the severity of PTSD symptoms, the type of trauma experienced, or the duration since trauma.

*Comorbidities*

Studies with participants who were selected on the basis of having an existing diagnosis of PTSD or on the basis of attending a traumatic stress specific service will not be included. There will be no limitation on comorbidities of other disorders.

*Setting*

There will be no limitation on the study setting other than secondary care mental health services (or services specifically for mental health disorders if primary/secondary care distinction does not exist in that country).

***Types of outcome measures***

*Primary Outcomes*

1. Prevalence of undetected PTSD, as measured as a percentage based on the results of the previously discussed assessments.

*Secondary Outcomes*

1. Primary diagnosis as indicated by their diagnosis from DSM or ICD.
2. Severity of symptoms, using a standardised scale such as PCL, TSQ or IES.
3. Type of trauma as categorised by an independent reviewer.
4. Length of time since primary diagnosis as measured in years and months.
5. Level of involvement with mental health services
6. Veterans
7. Demographic information
8. Other relevant ones identified through searches / literature

**Search Methods for the Identification of Studies**

*Databases*

To identify the relevant articles we will systematically search the following databases: PsychINFO, Embase, PubMed, and PILOTS.

*[Amendment to protocol*: databases searched revised for search dated 22^nd^ August 2016]

*Electronic searches*

*[Amendment to protocol*: search terms revised for search dated 22^nd^ August 2016]

| PTSD | PTSD or "post-traumatic stress disorder*" or "posttraumatic stress disorder*" or "post traumatic stress disorder*" or "post trauma*" or trauma or traumatic or "stress disorder*" or "acute stress" or “acute stress disorder” or "stress reaction*" or peritraumatic or "stress disorders, traumatic" or “stress disorders, post-traumatic” or “traumatic stress” or “psychological trauma*” or “combat disorder*” or veteran* or war or “military psychiatry” or “combat stress” or “stress disorders, traumatic, acute” |
| --- | --- |
| Mental Illness | "mental illness" OR "mental disorder*" OR "psychiatric disorder*" OR "mental health" OR "bipolar disorder*" OR bipolar OR schizophrenia OR schizophrenic OR schizoaffective OR psychotic OR psychosis OR anxiety OR depression OR depressive OR "major depressive disorder*" OR MDD OR agoraphobia OR "panic disorder*" OR "seasonal affective disorder*" OR SAD OR "social anxiety" OR "social anxiety disorder*" OR "social phobia*" OR phobia* OR mania OR manic OR hypomania* OR "manic depress*" OR "generalised anxiety disorder*" OR "generalized anxiety disorder*" OR GAD OR "generalised anxiety" OR "generalized anxiety" OR OCD OR "obsessive compulsive disorder*" OR anorexi* OR bulimi* OR "anorexia nervosa" OR "bulimia nervosa" OR "binge eating disorder" OR BED OR "eating disorder*" OR pica OR rumination OR "borderline personality disorder*" OR BPD OR "personality disorder*" OR "multiple personality disorder*" OR "dissociative identity disorder*" OR "attention deficit hyperactivity disorder" OR ADHD OR "mental health disorder*" OR axis 1 OR axis 2 OR axis I OR axis II OR "severe mental illness" OR SMI OR "substance abuse" OR "substance disorder" OR "drug abuse" OR "alcohol abuse" OR somatoform OR dissociat* |
| Undetected | Undetect OR undetected OR unrecognised OR unrecognized OR undiagnosed OR undiagnozed OR untreated OR underdiagnosed OR underdiagnozed OR overlooked OR under-detected OR under-diagnosed OR under-diagnozed OR under-recognised OR under-recognized OR under-report OR "did not identify" OR unidentified OR "failure to diagnose" |

*Limits:* Human adults aged 16 or over, peer reviewed, and published.

*Reference Lists*

Of the articles already included in our systematic review, we will hand search their references for additional studies which meet our inclusion criteria.

**Selection of studies**

Titles and abstracts of the studies will be independently screened by two review authors (HC and AP / others as required). The studies will be identified and if there is uncertainty the whole article will be retrieved. Any disputes will be resolved through direction from the third reviewer (HM / others as required). The reasons for inclusion and exclusion of the studies will be noted.

**Data extraction**

Two members of the research team (HC and AP / others as required) will extract all outcomes from the included studies using our inclusion/exclusion criteria. Information gathered in a formulated data capture sheet will include study year, study population, sample size, settings, study design, demographic details of participants, prevalence of undetected PTSD, assessment tools, outcomes and where appropriate trauma specifications. A third reviewer (HM / others as required) will resolve any disagreements to reach a unanimous decision.

**Assessment of risk of bias**

Two review authors (HC and AP / others as required) will assess the risk of bias in the chosen studies independently, and any disagreements will be settled by the third author (HM / others as required). Bias to be assessed using Hoy et al instrument (Assessing risk of bias in prevalence studies). [*Amendment to protocol:* December 2015: instrument adapted for specifics of this review, and key criteria for assessing bias selected]

**Data Synthesis and Analysis**

Meta-analysis of proportions of undetected PTSD across studies and provision of meta-analytic estimate if heterogeneity acceptable

Meta-regression of variables considered to be candidates for explaining heterogeneity is such heterogeneity is present
