# Supplementary material for: Undetected post-traumatic stress disorder in secondary-care mental health services: systematic review
Source: Br J Psychiatry. 2018 Jan;212(1):11–8. doi: 10.1192/bjp.2017.8 (PMC6457163; doi:10.1192/bjp.2017.8)
Supplement: Supplementary file 1 [file S0007125017000083sup001.zip › editedZammit et al online supplement DS3.docx]

**Search strategies (final): undiagnosed PTSD** 2016-08-22

OVID MEDLINE Epub Ahead of Print, In-Process & Other Non-Indexed Citations, Ovid MEDLINE(R) Daily and Ovid MEDLINE(R) 1946 to Present

1. stress disorders, traumatic/ or combat disorders/ or psychological trauma/ or stress disorders, post-traumatic/ or stress disorders, traumatic, acute/

2. (PTSD or posttrauma* or post trauma*).ti,ab,kf.

3. or/1-2

4. (hidden or undetect* or un detect* or underdetect* or under detect* or undocument* or un document* or unreport* or un report* or underreport* or under report* or unnoticed or un noticed or undiagnos* or un diagnos* or underdiagnos* or under diagnos* or without diagnosis or (miss* adj1 diagnos*) or unrecogni* or un recogni* or under recog* or underrecog* or unrepresent* or un represent* or underrepresent* or under represent* or (fail* adj3 (diagnos* or detect* or identif*)) or overlook*).ti,ab,kf.

5. ("not diagnosed" or "did not identify" or "was not identified").ab.

6. (improv* recognition or (increase* adj3 (assessment* or diagnos* or detect* or identif*))).ti,ab,kf.

7. or/4-6

8. exp mental disorders/ or exp anxiety disorders/ or exp "bipolar and related disorders"/ or exp "disruptive, impulse control, and conduct disorders"/ or exp dissociative disorders/ or exp elimination disorders/ or exp "feeding and eating disorders"/ or exp mood disorders/ or exp neurocognitive disorders/ or exp neurotic disorders/ or exp paraphilic disorders/ or exp personality disorders/ or exp "schizophrenia spectrum and other psychotic disorders"/ or exp somatoform disorders/ or exp substance-related disorders/

9. (bipolar or schizophreni* or schizoaffective or (psychotic adj2 (illness or disorder*)) or psychosis or smi or ((severe or serious*) adj2 mental*) or mania or manic or hypomani* or mood or depression or depressive or mdd or affective disorder* or anxiety disorder* or adnos or agoraphobi* or panic or social anxiety or phobi* or gad or obsessive compulsive or ocd or anorexi* or bulimi* or binge or binging or eating disorder* or ednos or pica or rumination or personality disorder* or bpd or dissociative disorder or hyperactivity disorder or adhd or substance abuse or substance use* or substance disorder or sud or suds or drug abus* or alcohol abuse or alcoholi* or chemical dependency or addict* or (cannabis adj2 (use* or abuse)) or somatoform or somati* or psychosoma* or trichotillomania or impulse control or selfharm* or self harm* or suicid* or parasucid*).ti,ab,kf.

10. (non-PTSD or axis-i or (mental* adj (ill* or health)) or psychiatric).ti,ab,kf.

11. or/8-10

12. 3 and 7 and 11

13. PREVALENCE/

14. prevalen*.ti,ab,kf.

15. screen*.ti,ab,kf.

16. or/13-15

17. 3 and 11 and 16

18. 12 or 17 *[n=7001, 2016-08-22]*

OVID PsycINFO (1987 to July Week 4 2016)

1. exp posttraumatic stress disorder/ or acute stress disorder/ or post-traumatic stress/ or traumatic neurosis/

2. (PTSD or posttrauma* or post trauma*).ti,ab,id.

3. 1 or 2

4. (hidden or undetect* or un detect* or underdetect* or under detect* or undocument* or un document* or unreport* or un report* or underreport* or under report* or unnoticed or un noticed or undiagnos* or un diagnos* or underdiagnos* or under diagnos* or without diagnosis or (miss* adj1 diagnos*) or unrecogni* or un recogni* or under recog* or underrecog* or unrepresent* or un represent* or underrepresent* or under represent* or (fail* adj3 (diagnos* or detect* or identif*)) or overlook*).ti,ab,id.

5. ("not diagnosed" or "did not identify" or "was not identified").ti,ab,id.

6. (improv* recognition or (increase* adj3 (assessment* or diagnos* or detect* or identif*))).ti,ab,id.

7. or/4-6

8. psychiatric patients/ or mental disorders/ or adjustment disorders/ or exp affective disorders/ or alexithymia/ or autism spectrum disorders/ or exp chronic mental illness/ or exp dementia/ or exp dissociative disorders/ or exp eating disorders/ or elective mutism/ or exp factitious disorders/ or exp gender identity disorder/ or exp hoarding disorder/ or exp hysteria/ or exp impulse control disorders/ or koro/ or mental disorders due to general medical conditions/ or exp neurosis/ or exp paraphilias/ or exp personality disorders/ or pseudodementia/ or exp psychosis/ or schizoaffective disorder/

9. anxiety disorders/ or castration anxiety/ or death anxiety/ or generalized anxiety disorder/ or exp obsessive compulsive disorder/ or panic disorder/ or exp phobias/ or separation anxiety disorder/

10. (non-PTSD or axis-i or (mental* adj (ill* or health)) or psychiatric).ti,ab,id.

11. or/8-10

12. prevalen*.ti,ab,id.

13. Screening/

14. screen*.ti,ab,id.

15. or/12-14

16. 3 and 7 and 11

17. 3 and 11 and 15

18. 16 or 17 *[n=3473]*

OVID EMBASE (1974 to 22-August-2016)

1. posttraumatic stress disorder/

2. acute stress disorder/

3. (PTSD or posttrauma* or post trauma*).ti,ab,kw.

4. or/1-3

5. (hidden or undetect* or un detect* or underdetect* or under detect* or undocument* or un document* or unreport* or un report* or underreport* or under report* or unnoticed or un noticed or undiagnos* or un diagnos* or underdiagnos* or under diagnos* or without diagnosis or (miss* adj1 diagnos*) or unrecogni* or un recogni* or under recogni* or underrecogni* or unrepresent* or un represent* or underrepresent* or under represent* or (fail* adj3 (diagnos* or detect* or identif*)) or overlook*).ti,ab,kw.

6. ("not diagnosed" or "did not identify" or "was not identified").ab.

7. (improv* recognition or (increase* adj3 (assessment* or diagnos* or detect* or identif*))).ti,ab,kw.

8. or/5-7

9. (non-PTSD or axis-i or (mental* adj (ill* or health)) or psychiatric).ti,ab,kw.

10. anxiety disorder/ or anxiety neurosis/ or cardiac anxiety/ or catastrophizing/ or distress syndrome/ or generalized anxiety disorder/ or koro/ or "mixed anxiety and depression"/ or obsessive compulsive disorder/ or panic/ or exp phobia/ or psychasthenia/ or separation anxiety/

11. mental disease/ or exp addiction/ or adjustment disorder/ or alexithymia/ or exp autism/ or exp behavior disorder/ or exp delirium/ or exp dementia/ or exp dissociative disorder/ or exp learning disorder/ or exp memory disorder/ or exp mental deficiency/ or mental infantilism/ or mental instability/ or mental overstimulation/ or exp mood disorder/ or exp neurosis/ or personality disorder/ or psychosexual disorder/ or psychosis/ or psychosomatic disorder/

12. (bipolar or schizophreni* or schizoaffective or (psychotic adj2 (illness or disorder*)) or psychosis or smi or ((severe or serious*) adj2 mental*) or mania or manic or hypomani* or mood or depression or depressive or mdd or affective disorder* or anxiety disorder* or adnos or agoraphobi* or panic or social anxiety or phobi* or gad or obsessive compulsive or ocd or anorexi* or bulimi* or binge or binging or eating disorder* or ednos or pica or rumination or personality disorder* or bpd or dissociative disorder or hyperactivity disorder or adhd or substance abuse or substance use* or substance disorder or sud or suds or drug abus* or alcohol abuse or alcoholi* or chemical dependency or addict* or (cannabis adj2 (use* or abuse)) or somatoform or somati* or psychosoma* or trichotillomania or impulse control or selfharm* or self harm* or suicid* or parasucid*).ti,ab,kw.

13. or/9-12

14. 4 and 8 and 13 *[903]*

15. PREVALENCE/

16. prevalen*.ti,ab,kw.

17. SCREENING/

18. screen*.ti,ab,kw.

19. or/15-18

20. 4 and 13 and 19 *[7652]*

21. limit 20 to exclude medline journals [735]

22. (14 or 21) *[1595]*

**ProQuest PILOTS: Published International Literature On Traumatic Stress** 17-Aug-2016

*Search 1*: ((screen* near/3 (PTSD or posttrauma* or "post trauma*") OR (diagnos* near/3 (PTSD or posttrauma* or "post trauma*") OR ([prevalen* near/3 (PTSD or posttrauma* or "post trauma*")](http://search.proquest.com/recentsearches.recentsearchtabview.recentsearchesgridview.scrolledrecentsearchlist.checkdbssearchlink:rerunsearch/7DCA0303F96C4777PQ/None?site=pilots&t:ac=RecentSearches))) AND (ti(non-PTSD or axis-1 or "mental illness" or "mentally ill" or "mental health" or "mental disorder*" or psychiatric) OR ti((bipolar or schizophreni* or schizoaffective or psychotic or psychosis or smi or “severe mental” or “serious mental” or mania or manic or hypomani* or mood or depression or depressive or mdd or "affective disorder*" or "anxiety disorder*" or adnos or agoraphobi* or panic or "social anxiety" or phobi* or gad or "obsessive compulsive" or ocd or anorexi* or bulimi* or binge or binging or "eating disorder*" or ednos or pica or rumination or "personality disorder*" or bpd or "dissociative disorder" or "hyperactivity disorder" or adhd or "substance abuse" or "substance use*" or "substance disorder" or sud or suds or "drug abus*" or "alcohol abuse" or alcoholi* or "chemical dependency" or addict* or “cannabis use*” or “cannabis abuse” or somatoform or somati* or psychosoma* or trichotillomania or "impulse control" or selfharm* or "self harm*" or suicid* or parasucid*))

*Search 2*: ((PTSD or posttramatic or "post traumatic") AND (“not diagnosed” or "did not identify" or "was not identified" or undetect* or “un detect*” or underdetect* or “under detect*” or undocument* or “un document*” or unreport* or “un report*” or underreport* or “under report*” or unnoticed or “un noticed” or undiagnos* or “un diagnos*” or underdiagnos* or “under diagnos*” or “without diagnosis” or “miss* diagnos*” or unrecogni* or “un recogni*” or unrepresent* or “under recogni*” or underrecogni* or “un represent*” or underrepresent* or “under represent*” or overlook* OR (fail* near/3 (diagnos* or detect* or identif*)))

*[n=1537]*

**Authors emailed for further information to help determine inclusion of study**

Dr M. Bonn-Miller

Bonn-Miller, M. O., et al. (2012). "The underdiagnosis of cannabis use disorders and other Axis-I disorders among military veterans within VHA." Military medicine **177**(7): 786-788

Dr K. Cusack

Cusack, K. J., et al. (2006). "Unrecognized trauma and PTSD among public mental health consumers with chronic and severe mental illness." Community Mental Health Journal **42**(5): 487-500.

Dr P. de Bont / Dr M. van den Berg

de Bont, P. A., et al. (2015). "Predictive validity of the Trauma Screening Questionnaire in detecting post-traumatic stress disorder in patients with psychotic disorders." Br J Psychiatry **206**(5): 408-416.

Dr N. Gielen

Gielen N, Havermans RC, Tekelenburg M, et al. Prevalence of post-traumatic stress disorder among patients with substance use disorder: it is higher than clinicians think it is. European journal of psychotraumatology 2012;**3** doi: 10.3402/ejpt.v3i0.17734

Dr V. Gosein / Dr E. Ford

Gosein VJ, Stiffler JD, Frascoia A, Ford EB. *J Forensic Sci*. **2016** Jan;61(1):116-21. Life Stressors and Posttraumatic Stress Disorder in a Seriously Mentally Ill Jail Population.

Dr O. Kavakci

Kavakci O, et al. (2013). "Prevalence of post-traumatic stress disorder among the inpatients in a tertiary clinic and relationship with suicidal attempts." Neurology psychiatry and brain research **19**: 76-79.)

Dr A. Komiti

Komitit et al (2001). A comparison of the Composite International Diagnostic Interview (CIDI-Auto) with clinical assessment in diagnosing mood and anxiety disorders. *Australian and New Zealand journal of psychiatry* **35**, 224-230.

Dr A. McFarlane

McFarlane, A., et al. (2001). "Posttraumatic Stress Disorder in a General Psychiatric Inpatient Population." Journal of Traumatic Stress **14**(4): 633-645.

Dr D. Stein / Dr R. Kessler

Stein DJ, Chiu WT, Hwang I, Kessler RC, Sampson N, Alonso J, et al. (2010) Cross-National Analysis of the Associations between Traumatic Events and Suicidal Behavior: Findings from the WHO World Mental Health Surveys. PLoS ONE 5(5): e10574. doi:10.1371/journal.pone.0010574

Dr S. Tagay

Tagay, S., et al. (2005). "Posttraumatic stress disorder in a psychosomatic outpatient clinic: Gender effects, psychosocial functioning, sense of coherence, and service utilization." Journal of Psychosomatic Research **58**(5): 439-446.

Tagay, S., et al. (2010). "Traumatic events, posttraumatic stress symptomatology and somatoform symptoms in eating disorder patients." European Eating Disorders Review **18**(2): 124-132.

Dr B. Wang

Wang, B. and S. Vivek (2013). "Survey of posttraumatic stress disorder (PTSD) with PTSD Checklist - Civilian (PCL-C) Questionnaire on outpatients at two mental health clinics in New York City." Journal of Depression and Anxiety S4.
