# Supplementary material for: Undetected post-traumatic stress disorder in secondary-care mental health services: systematic review
Source: Br J Psychiatry. 2018 Jan;212(1):11–8. doi: 10.1192/bjp.2017.8 (PMC6457163; doi:10.1192/bjp.2017.8)
Supplement: Supplementary file 1 [file S0007125017000083sup001.zip › editedZammit et al online supplement DS4.docx]

Online Supplement DS4: Study Eligibility Form

| FACTORS | ASSESSMENT | COMMENTS |
| --- | --- | --- |
| Type of study |  |  |
| 1. Has the study been peer reviewed and is it already published?  NB: Both must apply. | Yes Unclear No:    **Exclude** |  |
| 2. Was the study published after 1980? | Yes Unclear No:    **Exclude** |  |
| Participants |  |  |
| 3. Do the participants currently suffer from a mental illness (excluding PTSD) – i.e. specifically recruited through secondary care/psychiatric services?  NB: Diagnosed according to DSM-III, DSM-IIIR, DSM-IV, DSM-V, ICD-9, ICD-10 or via a clinical diagnosis.  NB: if all participants selected on basis of having diagnosis of PTSD or on basis of attending a traumatic stress service then answer “No”. | Yes Unclear No:    **Exclude** |  |
| 4. Are patients screened for current PTSD?  NB: PTSD diagnosed using a structured interview or using a DSM/ICD based tool (e.g. Impact of Event Scale, Trauma Screening Questionnaire etc.) | Yes Unclear No:    **Exclude** |  |
| 5. Were the participants aged 16 or over?  NB: If mixed & subgroup data available for age >16, or if >75% of sample age >16 answer ’yes’ | Yes Unclear No:    **Exclude** |  |
| Outcomes |  |  |
| 6. Does the study report N or % of participants with current PTSD on screening, and N or % with diagnosis of PTSD in clinical records?  NB: Answer “Yes” if the paper reports undiagnosed PTSD result as a usable figure in participants who had a pre-existing mental illness. | Yes Unclear No:    **Exclude** |  |
