# Supplementary material for: Undetected post-traumatic stress disorder in secondary-care mental health services: systematic review
Source: Br J Psychiatry. 2018 Jan;212(1):11–8. doi: 10.1192/bjp.2017.8 (PMC6457163; doi:10.1192/bjp.2017.8)
Supplement: Supplementary file 1 [file S0007125017000083sup001.zip › editedZammit et al online Tables and Fig.docx]

**Table DS1** Quality of studies

| **Study** | **Random or consecutive sampling^1^** | **Response rate reported^1^** | **Response rate 75% or more^1^** | **Blinding of screening^2^ / records^1^** | **Overall risk of bias^4^** | **Other bias** |
| --- | --- | --- | --- | --- | --- | --- |
| **Alvarez 2012** | No | No | - | Yes/No | High | *Possible* *underestimate* of undetected PTSD due to BPRS exclusion criterion |
| **Bonn-Miller 2012** | No | No | - | No/No | High | - |
| **Brady 2003** | No | No | - | No/Yes | High | *Possible* *underestimate* of undetected PTSD as ‘PTSD’ in notes includes any anxiety disorder + mention of trauma history |
| **Calhoun 2007** | Yes | Yes | Yes | Yes/No | Low | - |
| **Cascardi 1996** | No | Yes | No (60%) | Yes/No | High | *Possible* *underestimate* of undetected PTSD as only screened if experienced aggression |
| **Craine 1988** | Yes | No | No | No/No | Medium | - |
| **Cusack 2006** | Yes | Yes | Yes | Yes/No | Low | - |
| **Dansky 1997** | No | No | - | No/No | High | *Possible* *underestimate* of undetected PTSD as results of PTSD screening was recorded in notes (and communicated with clinical team if salient) |
| **Davidson 1990** | Yes | Yes | Yes | No/No | Low | - |
| **de Bont 2015** | Unclear | No | - | Yes/No | High | - |
| **Gielen 2012** | Yes | Yes | Yes | Yes/No | Low | - |
| **Gosein 2016** | Yes | Yes | No (60%) | No/No | Medium | - |
| **Howgego 2005** | Yes | Yes | No (32%) | No/No | Medium | - |
| **Kavakci 2013** | Yes | No | - | Yes/No | Medium | - |
| **Kilcommons 2005** | No | No | - | Yes/No | High | - |
| **Kimerling 2006** | No | No | - | No/No | High | - |
| **Komiti 2001** | Yes | Yes | Yes | No/No | Low | - |
| **Lommen 2009** | Yes | Yes | No (44%) | Yes/No | Medium | - |
| **McFarlane 2006** | Yes | Yes | No (31%) | No/No | Medium | - |
| **Mueser 1998** | No | Yes | Yes | Yes/No | Medium | - |
| **Reynolds 2005** | Yes | No | - | Yes/No | Medium | - |
| **Reynolds 2011** | No | Yes | No (60%) | Yes/No | Medium | - |
| **Schwartz 2005** | No | Yes | Yes | No/No | Medium | - |
| **Switzer 1999** | No | Yes | Yes | Yes/Yes | Medium | - |
| **Tagay 2005** | No | Yes | Yes | Yes/No | Medium | Level of severity used to define cut-off for PTSD on screening is unclear |
| **Tagay 2010** | Yes | No | - | Yes/No | Medium | Level of severity used to define cut-off for PTSD on screening is unclear |
| **Van Zyl 2008** | Yes | No | - | No/Yes | Medium | - |
| **Villano 2007** | No | Yes | No (51%) | No/No | High | - |
| **Wang 2013** | No | No | - | Yes^3^/No | High | - |

Footnote: ^1^ Coded as 1 if ‘Yes’; 0 if ‘No’ or ‘Unclear’; ^2^ Coded as 1 if ‘Yes’ or self-report measure used; 0 if ‘No’ or ‘Unclear’; ^3^ Blinding assumed as stated that clinical records viewed after PTSD screening had been done; ^4^ High risk (sampling not random/consecutive and <75% response), Medium (sampling not random/consecutive or <75% response), Low risk (sampling random/consecutive and ≥75% response)

Online Table DS2 Description of included studies

| **Study** | **Country** | **Sample** | **Diagnoses** | **N** | **Mean age** | **Male (%)** | **PTSD**  **screen** | **PTSD**  **criteria** | **N (%) PTSD on screen** | **N (%) PTSD in records** | **Undetected PTSD^1^** |
| --- | --- | --- | --- | --- | --- | --- | --- | --- | --- | --- | --- |
| **Alvarez**  **2012^37^** | Spain | Mental Health Centre | SCZ 51%  BP 39.2%  SA 9.8% | 102 | 39 | 53 | TLEQ & DEQ | DSM-IV  (A-F) | 14 (15.1%) | 2 of the 14 | 11.8% |
| **Bonn-Miller 2012^17^** | USA | Veterans from mental health clinics in VA Medical Centre | CUD | 84 | 52 | 96 | CAPS | DSM-IV | 31 (36.9%) | 18 (21.4%) | 15.5% |
| **Brady**  **2003^10^** | USA | Urban community mental health centre | SCZ/SA 77%  MD 23%  42% also SUD | 64 | 42 | 48 | SCID | DSM-IV | 15 (23%) | 2 (3%) | 0.3% |
| **Calhoun**  **2007^18^** | USA | Veterans in inpatient psychiatric unit | SCZ | 165 | 48 | 100 | PCL-17 | DSM-IV decision rule | 78 (47%) | 11 (14%) | 40.6% |
| **Cascardi**  **1996^19^** | USA | Psychiatric  inpatients | SCZ 29%  AffD 60% | 69 | 33 | 49 | PSS | DSM-IV symptom criteria | 20 of 42 screened (48%) | 0 | 29% |
| **Craine**  **1988^20^** | USA | Psychiatric  inpatients | SCZ 41%  AffD 22%  PD 22%  AdjD 11% | 105 | 35 | 0 | Structured interview | DSM-III | 36 (34.3%) | 0 | 34.3% |
| **Cusack**  **2006^21^** | USA | Psychiatric outpatients | SMI | 142 | 46 | 56 | PCL-17 | Cut-off 50^2^  Cut-off 45  DSM-IV based score method | 27 (19%)  42 (29.6%)  43 (30.3%) | 5 (3%) | 15.5%  26.1%  26.8% |
| **Dansky**  **1997^22^** | USA | Psychiatric  inpatients | SUD | 95 | 35 | 36 | NWS PTSD Module | DSM-III | 42 (44.2%) | 13 (13.7) | 30.5% |
| **Davidson 1990^11^** | USA | Psychiatric outpatients | AdjD ~30%  AffD ~26%  AnxD ~26%  PD ~17% | 54 | 33 | 39 | Clinical interview | DSM-III | 7 (13%) | 1 (1.9%) | 11.1% |
| **de Bont**  **2015^32^** | Netherlands | Outpatients in long-term mental health care services | PsyD | 2608 | 42 | 62 | TSQ | cut-off^2^ 6+  Predictive model^3^ | 743 (28.5%)  416 (16%) | 13 (0.5%) | 28.0% |
| **Gielen**  **2012^33^** | Netherlands | Addiction treatment centres | SUD | 423 | 41 | 79 | TEC & SRIP | DSM-IV  (A-D) | 156 (36.9%) | 9 (2.1%) | 34.8% |
| **Gosein**  **2016^23^** | USA | Incarcerated patients in psychiatric hospital | PsyD 54%  MD 21%  AdjD 23% | 48 | 34 | 100 | SCID-I | DSM-IV | 22 (46%) | 1 (2%) | 43.8% |
| **Howgego 2005^27^** | Australia | Community mental health service | SCZ 59%  BPD 19%  MDD 11%  BP 11% | 27 | 37 | 52 | PDS  Interviewer administered | DSM-IV  (A-F) | 9 (33%) | 1 (3.7%) | 29.6% |
| **Kavakci**  **2013^38^** | Turkey | Psychiatric  inpatients | BP 22%  SCZ+SA 22%  MDD 21% | 175 | 35 | 45 | PDS | DSM-IV  (A-F?) | 53 (30.3%) | 3 (1.7%) | 28.6% |
| **Kilcommons 2005^6^** | UK | Community mental health team | PsyD | 32 | 35 | 78 | THQ & PSS-SR | DSM-IV  (A-E) | 17 (53%) | 1 (3.1%) | 50% |
| **Kimerling 2006^7^** | USA | VA Substance Use Treatment Clinics | SUD | 97 | 48 | 98 | CAPS | DSM-IV | 32 (33%) | 8 of 32  (25%) | 49.7% |
| **Komiti**  **2001^28^** | Australia | Anxiety & Mood Disorders outpatients | MDD 18.6%  AnxD 60.4% | 262 | 35 | 33 | CIDI | DSM-IV | 29 (11.1%) | 6 (2.3%) | 23/262 = 8.8% |
| **Lommen 2009^34^** | Netherlands | Psychiatric outpatients | SCZ 70%  SA 30% | 33 | 42 | 70 | PSS-SR | DSM-IV (A-F)  A1 criterion  (+/-) & symptoms ≥ twice/week (+/-) | A1+ Sym +  3 (9.1%)^2^  A1+ Sym -  6(18.2%)  A1- Sym +  7(21.2%)  A1- Sym -  13(39.4%) | 0 | 9.1%  18.2%  21.2%  39.4% |
| **McFarlane 2006^29^** | Australia | Psychiatric  inpatients | PsyD 40&  MDD 25%  BD 17%  AdjD 15% | 130 | 37 | 53 | CIDI | DSM-IV | 36 (27.7%) | 7 (5.4%) | 22.3% |
| **Mueser**  **1998^8^** | USA | Psychiatric  inpatients and outpatients | SCZ 23%  SA 11%  BD 18%  MDD 24%  BPD 8% | 275 | 40 | 44 | THQ & PCL-S | DSM-IV  (A-D) | 119 (43.3%)  SCZ 18/64 (28%)  SA 11/30  (37%)  BP 20/50 (40%)  MDD 38/65  (58%)  BPD 12/22 (54%) | 3 of 119 | 42.2%  Lower-limit:  SCZ 15/64 (23.4%)  SA 8/30 (26.7%)  BP 17/50 (34%)  MDD 35/65 (53.8%)  BPD 9/22 (41%) |
| **Reynolds 2005^30^** | UK | Addiction services inpatients | SUD | 52 | 34.6 | 59.6 | PSS-I | DSM_IV (A-F) | 20 (38.5%) | 1 (0.02%) | 36.5% |
| **Reynolds 2011^31^** | UK | Community drug and alcohol service | SUD | 42 | 36.4 | 64.3 | PSS-I | DSM_IV (A-F) | 11 (26.2%) | 0 | 26.2% |
| **Schwartz 2005^24^** | USA | Psychiatric outpatients | SMI | 66 | 45 | 36 | SCID-I | DSM-IV | 26 (39.4%) | 3 of 26 (4.5%) | 34.8% |
| **Switzer**  **1999^25^** | USA | Psychiatric outpatients | SUD 48%  MDD 39%  SCZ-s 24% | 181 | - | 26 | CIDI | DSM-III-R | 72 (39.8%) | 2 of 72 (1.1%) | 38.7% |
| **Tagay**  **2005^12^** | Germany | Psycho-somatic Medicine Clinic outpatients | ED 34.5%  MDD 20.6%  AnxD 17%  SomD 13.7% | 583 | 35 | 26 | PDS | DSM-IV  (A criteria + IES-R cut-off)  IES-R cut-off | 59 (10.1%)  91 (15.6%) | 16 (2.7%) | 7.4%  12.9% |
| **Tagay**  **2010^35^** | Germany | Psycho-somatic Medicine Clinic outpatients | ED | 101 | 27 | 0 | PDS | DSM-IV  (A criteria + IES-R cut-off) | 13 (12.9%) | 3 of 13 (3%) | 9.9% |
| **Van Zyl**  **2008^36^** | South Africa | Anxiety & mood disorders Unit inpatients | MDD 44%  AdjD 13%  BP I 13%  BP II 19%  OMD 13% | 40 | 36 | 18 | CAPS | DSM-IV | 16 (40%) | 0 | 40% |
| **Villano**  **2007^26^** | USA | Mental health clinic outpatients | SCZ 28%  MDD 16%  BP 14%  OMD 26% | 295 | 39 | 59 | MINI | DSM-IV | 135 (46%) | 21 (7%) | 38.6% |
| **Wang**  **2013^9^** | USA | Mental health clinic outpatients | SCZ 30%  BP 32%  MDD 27%  SUD 22% | 62 | 45 | 36 | TLEQ & PCL-C | DSM-IV  (A criteria + cut-off 44) | 38 (61%) | 8 (13%) | 48.4% |

**Footnote:** ^1^Undetected PTSD (% of sample); ^2^ Used for meta-analysis; ^3^ based on TSQ, sexual abuse, physical abuse, severe neglect. We are grateful for the following authors for providing additional information about their study: Dr K. Cusack, Dr E. Ford, Dr N. Gielen, Dr. A. Komiti, Dr. S. Tagay, Dr B. Wang

Abbreviations: AdjD Adjustment disorder; AffD Affective disorder; AnxD Anxiety disorder; BP Bipolar disorder; BPD Borderline Personality Disorder; BPRS Brief Psychiatric Rating Scale; CAPS Clinician-administered PTSD scale; CIDI Composite international diagnostic interview; CUD Cannabis use disorder; DEQ Distressing Event Questionnaire; IES-R Impact of Events Scale Revised; MD Mood disorder; MDD Major depressive disorder; MINI Mini-international psychiatric interview; NWS National Women’s Study; OMD Other mood disorder; PCL-17 PTSD checklist (17-item version); PCL-C PTSD checklist (Civilian); PCL-S PTSD checklist (Specific); PD Personality disorder; PDS Post-traumatic stress diagnostic scale; PSS PTSD Symptom Scale; PSS-SR PTSD Symptom Scale – self report; PSS-SR PTSD Symptom Scale – interview; PsyD Psychotic disorder; SA Schizo-affective disorder ; SCID-I Structured clinical interview for DSM-IV Disorders; SCZ Schizophrenia; SCZ-s Schizophrenia-spectrum; SMI Severe mental illness; SomD Somatoform Disorder; SRIP Self-Rating Inventory for Post-traumatic stress disorder; SUD Substance use disorder; TEC Trauma Exposure checklist; THQ Trauma History Questionnaire; TLEQ Traumatic Life Events Questionnaire; TSQ Trauma Screening Questionnaire; VA Veterans Affairs

**Supplementary Figure DS1:** Prevalence of PTSD on screening

ES = Effect size (proportion)

**Supplementary Figure DS2:** Prevalence of PTSD on screening, by primary diagnostic sub-category

ES = Effect size (proportion)

**Figure DS3:** Proportion of sample with undetected PTSD, by diagnostic sub-group (where 50% or more of sample are in the same diagnostic subgroup)

ES = Effect size (proportion)

**Table DS3** Meta-regression of explanatory variables for PTSD outcomes

|  | **PTSD on screening** | | |  | **Detection of PTSD** | | |  | **Undetected PTSD** | | |
| --- | --- | --- | --- | --- | --- | --- | --- | --- | --- | --- | --- |
| *Variable^1^* | *Beta* | *95% CI* | *P* |  | *Beta* | *95% CI* | *P* |  | *Beta* | *95% CI* | *P* |
| **Year** | 0.01 | -0.07, 0.09 | 0.739 |  | -0.01 | -0.10, 0.07 | 0.717 |  | 0.00 | -0.08, 0.08 | 0.928 |
| **Age** | 0.08 | -0.01, 0.18 | 0.078 |  | 0.06 | -0.07, 0.19 | 0.350 |  | 0.04 | -0.05, 0.14 | 0.334 |
| **Percent male** | 0.02 | -0.00, 0.04 | 0.071 |  | 0.01 | -0.02, 0.03 | 0.660 |  | 0.01 | -0.01, 0.03 | 0.207 |
| **Country** | 0.13 | 0.04, 0.22 | 0.007 |  | 0.08 | -0.02, 0.18 | 0.126 |  | 0.09 | -0.01, 0.19 | 0.050 |
| **Veterans** | 0.10 | -0.08, 0.27 | 0.263 |  | 0.18 | -0.00, 0.37 | 0.053 |  | 0.01 | -0.16, 0.18 | 0.886 |
| **Inpatients** | 0.05 | -0.01, 0.11 | 0.106 |  | -0.02 | -0.09, 0.06 | 0.685 |  | 0.05 | -0.01, 0.11 | 0.072 |
| **Psychosis** | 0.77 | -0.82, 2.35 | 0.331 |  | -1.32 | -2.45, -0.18 | 0.024 |  | 1.05 | -0.43, 2.53 | 0.156 |
| **PTSD tool** | -0.03 | -0.14, 0.08 | 0.591 |  | -0.10 | -0.21, 0.01 | 0.068 |  | -0.01 | -0.11, 0.10 | 0.917 |
| **Low bias risk** | -0.04 | -0.11, 0.03 | 0.272 |  | -0.01 | -0.09, 0.07 | 0.748 |  | -0.02 | -0.09, 0.05 | 0.545 |

^1^ Year - per 10 year increase; Age - per 10 year increase in mean age; Male - per 10% increase; Country – coded as USA (1) vs rest (0); Veterans – coded as veterans (1) versus non-veterans (0); Inpatients - coded as outpatients only (0), mixed (1), inpatients only (2); Psychosis - per 10% increase in sample with a psychotic disorder; PTSD tool – coded as interview (0) versus questionnaire (1); Low bias risk – coded as non-low (0), intermediate (1), low (2)
